# Mutation Profile of HPV16 L1 and L2 Genes in Different Geographic Areas

**DOI:** 10.3390/v15010141

**Published:** 2022-12-31

**Authors:** Dimitris Tsakogiannis, Marios Nikolaidis, Flora Zagouri, Eleni Zografos, Christine Kottaridi, Zaharoula Kyriakopoulou, Lamprini Tzioga, Panayotis Markoulatos, Grigoris D. Amoutzias, Garyfalia Bletsa

**Affiliations:** 1 Research Center, Hellenic Anticancer Institute, 10680 Athens, Greece; 2 Bioinformatics Laboratory, Department of Biochemistry and Biotechnology, University of Thessaly, 41500 Larissa, Greece; 3 Department of Clinical Therapeutics, Alexandra Hospital, National and Kapodistrian University of Athens School of Medicine, 11528 Athens, Greece; 4 Department of Genetics, Development and Molecular Biology, School of Biology, Aristotle University of Thessaloniki, 54124 Thessaloniki, Greece; 5 Department of Environment, School of Technology, University of Thessaly, Gaiopolis, 41500 Larissa, Greece

**Keywords:** HPV16, variability, L1, L2, epitopes, capsid proteins, vaccines

## Abstract

The causal relationship between HPV and cervical cancer in association with the high prevalence of high risk HPV genotypes led to the design of HPV vaccines based on the major capsid L1 protein. In recent years, capsid protein L2 has also become a focal point in the field of vaccine research. The present review focuses on the variability of HPV16 L1 and L2 genes, emphasizing the distribution of specific amino acid changes in the epitopes of capsid proteins. Moreover, a substantial bioinformatics analysis was conducted to describe the worldwide distribution of amino acid substitutions throughout HPV16 L1, L2 proteins. Five amino acid changes (T176N, N181T; EF loop), (T266A; FG loop), (T353P, T389S; HI loop) are frequently observed in the L1 hypervariable surface loops, while two amino acid substitutions (D43E, S122P) are adjacent to L2 specific epitopes. These changes have a high prevalence in certain geographic regions. The present review suggests that the extensive analysis of the amino acid substitutions in the HPV16 L1 immunodominant loops may provide insights concerning the ability of the virus in evading host immune response in certain populations. The genetic variability of the HPV16 L1 and L2 epitopes should be extensively analyzed in a given population.

## 1. Introduction

Human Papillomaviruses (HPVs) are non-enveloped, double–stranded circular DNA viruses of 8 kb in size that infect basal keratinocytes of mucosal and cutaneous epithelia [1,2]. Based on the sequence similarity of the L1 gene, HPVs are grouped into five genera including Alphapapillomaviruses (alpha), Betapapillomaviruses (beta), Gammapapillomaviruses (gamma), Mupapillomaviruses (mu), and Nupapillomaviruses (nu) [3,4]. HPV genomes are further classified into intratypic variants according to the diversity of their genome sequence. Members of the same lineage display a nucleotide divergence of 1% to 10%, whereas members of the same sub-lineage display a nucleotide divergence of 0.5% to 1% [5,6,7,8]. Considering their carcinogenic potential, mucosal alpha-PVs are designated as high-risk (HR-HPV) and low-risk (LR-HPV) [3,4]. Currently, 15 HPV genotypes have been described as high-risk (HPV16, 18, 31, 33, 35, 39, 45,51, 52, 56, 58, 59, 68, 73 and 82), and 12HPV genotypes have been characterized as low-risk (HPV6, 11, 40, 42, 43, 44, 54, 61, 70, 72, 81, and CP6108) [9]. Long-term HR-HPV infection is the leading cause of cervical cancer development with HPV16 and HPV18 being identified in 70% of cervical cancer incidences worldwide [10]. It is considerable to highlight that HPV infection is also responsible for the high rates of vulvar, vaginal, anal, penile, and oropharyngeal cancers [11].

HPVs infect the basal layer of the epithelium via micro-wounds [12,13]. To initiate viral infection, the L1 capsid protein binds to herparin sulfate proteoglycans (HSPGs) located on the epithelial cell surface or on the basement membrane or it can interact with laminin-332 on the extracellular matrix [14,15]. After binding, the viral capsid undergoes cyclophilin (CyP) B-mediated conformational modifications that eventually expose the minor capsid protein L2 [14,15]. The binding of HPV to a secondary receptor is necessary for the effective viral insertion [14,16]. After viral entry the replication of HPV DNA is associated with the epithelial differentiation program [17]. First an initial replication phase preserves a stable number of viral episomes (50–100 per cell). Second, during the latent infection, the copy number of viral DNA remains stable and the viral genome is replicated along with the host cell DNA. Third, during the productive infection that occurs in the middle layers of the epitheliun the viral copy number increases guided by the different functions of E6, E7, E1 and E2 early proteins [18,19]. Over the late stage of viral infection that takes place in the upper layers of the epithelium, the viral capsid L1, L2 proteins form the new viral capsids, while the E4 protein triggers the total collapse of the epithelial cell intermediate filament, facilitating the release of newly generated viruses [20,21].

HPVs have established molecular mechanisms in order to prevent cell-cycle arrest and apoptosis. The viral E6 and E7 oncoproteins are involved in the uncontrolled cell proliferation as they inactivate tumor suppressor proteins p53 and pRB, respectively [22,23,24]. The E6 oncoprotein generates a complex with the E3 ubiquitin ligase E6-associated protein (E6AP) that subsequently triggers the polyubiquitination of p53 via the ubiquitin-mediated degradation pathway [22]. Accordingly, the E7 oncorotein interacts with pRb and releases the E2F transcription factor from the pRB/E2F suppressor complex. The disruption of the pRB/E2F complex enables the stimulation of the mitotic phase of cell cycle and consequently triggers cell proliferation [25,26]. An additional mechanism that is strongly implicated in viral carcinogenicity is the integration of HPV DNA into the host genome. The integration of viral DNA can occur through disruption of the E1, E2, L2 or L1 genes, whereas the E6 and E7 genes always remain intact and integrated into the host chromosome [27,28,29]. The viral integration causes additional chromosomal damages and genome destabilization in the infected cells, whereas the E6 and E7 oncogenes are extensively expressed [28,30,31,32,33].

The genome of HPV16 is organized in three major regions; the long control region (LCR), the early region that encodes for the early genes E1, E2, E4, E5, E6 and E7, and the late region that encodes for the late genes L1 and L2 [27,28,34]. A large-scale evolutionary analysis enabled the classification of HPV16 into four major variant intratypic lineages (A–D) and 16 sub-lineages (A1–4, B1–4, C1–4 and D1–4) [6,7,35]. The prevalence of the different HPV16 lineages is associated with the geographic origin, ethnicity and tumorigenic capacity of viral DNA [6]. Specific nucleotide variations and amino acid substitutions have been extensively associated with specific HPV16 lineages, whereas particular mutations of HPV16 DNA have been extensively investigated and related with a higher risk of severe dysplasia and cervical cancer development, including E6: Q14H, H78Y, L83V, Ε7: N29S, S63F, E2: H35Q, P219S, T310K and E5: I65V [36]. Notably, a recent bioinformatics tool (HPV16-Genotyper) was designed to facilitate the rapid identification of these cancer-related mutations simplifying the entire computational process of HPV16 lineage genotyping and the detection of potential intratypic recombination events or genomic mis-assemblies due to co-infections [37].

Cervical cancer is the fourth most common type of cancer and the second leading cause of cancer-related death in women in developing countries [38]. Cytological testing (the Papanicolaou and the Pap test) and HPV screening have substantially reduced the incidence of cervical cancer; however, it is still a major global public health issue [28,39]. In developed countries, HPV vaccination programs have significantly reduced the incidences of HPV infection [40,41]. The prophylactic HPV vaccines are produced based on the ability of HPV L1 major capsid proteins to be self-organized into empty capsid like structures, called virus-like particles (VLPs) [42,43]. HPV vaccines confer high immunogenicity due to structural characteristics of L1 VLPs which are responsible for the production of long-lasting antigen-specific antibody-generating cells [44,45,46]. HPV vaccines trigger the formation of neutralizing antibodies against the specific HPV vaccine genotypes [45,46]. Moreover, previous studies have proposed that intratypic HPV variants may exhibit novel epitopes specific to geographic locations and/or ethnic groups [47,48]. The accumulation of non-synonymous mutations in the L1 protein can modify protein structure and function and subsequently provide the virus with a selective advantage to escape immune response [47,48].

In the present study, we review the non-synonymous nucleotide variations that have been reported in the L1 and L2 capsid genes of HPV16 DNA with a special focus on those which are located in antigenic epitopes. Moreover, the impact of the L2 minor protein on prevention of viral transmission is also discussed. Finally, we performed a large-scale analysis of publicly available HPV16 L1 and L2 gene sequences in order to identify the global distribution of particular amino acid changes which may affect the aggression of the virus.

## 2. Types of HPV Vaccines and Mechanism of Antibody Protection

Currently, the US Food and Drug Administration (FDA) has approved three prophylactic HPV vaccines. In 2006, the FDA first approved Gardasil (Merck), which provides protection against four HPV genotypes (HPV 6, 11, 16, 18). Next, Cervarix (GlaxoSmithKline) was approved by the FDA in 2009, and it provides immunity against HPV16 and HPV18. Finally, in 2014 the FDA approved a supplemental use of Gardasil 9 (HPV nine—valent vaccine) expanding its administration in women and men of 27 to 45 years of age. Gardasil 9 protects against HPV 6, 11, 16, 18, 31, 33, 45, 52 and 58 [49]. The design of the currently available vaccines is based on virus-like particles (VLPs) that are generated by the major capsid protein L1 and mimics the structure of virions [50,51,52,53]. VLPs do not contain viral genome and they are considered safer than attenuated or inactivated viruses that could be turned into infectious ones [54,55].

**Figure 1:**
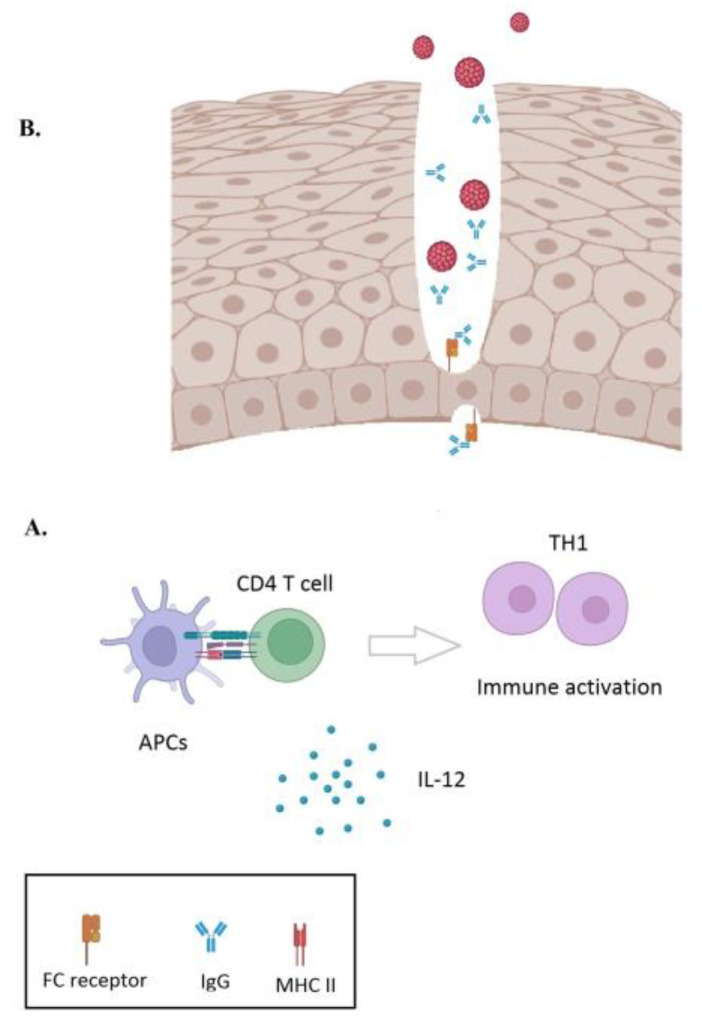
(**A**)After HPV vaccination, the antigen-presenting cells (APCs) expose the L1 epitopes to CD4^+^T cells through the major histocompatibility complex class II molecules (MHC-II). CD4^+^T cells in turn secrete cytokines that activate B-cells, T-cells (TH1; T-Helper 1 cells) and macrophages. HPV vaccination generates prolonged IgG antibody titers. (**B**) The produced antibodies (IgG) traverse the epithelial layer through the neonatal Fc receptor that is expressed at the epithelial cells of the cervix. In addition, IgG can also be transferred directly at the site of injury in the basal epithelial cell layer in order to block viral infection.

>The HPV VLPs have a highly ordered structure that exposes the L1 epitopes, promoting T-cell activation. In particular, the major histocompatibility complex class II molecules (MHC-II) that are expressed in the antigen-presenting cells (APCs) enable the presentation of the L1 epitopes to CD4^+^T cells. CD4^+^T cells in turn secrete cytokines, thus leading to the activation of B-cells, T-cells and macrophages [56,57,58,59] (Figure 1). HPV vaccination generates high-quality and prolonged antibody titers which are mostly immunoglobulin (Ig) G against the respective HPV L1 proteins [60,61]. Notably, HPV vaccination produces 10- to 100-fold higher titers of L1 specific neutralizing antibodies compared to natural infection, thus preventing viral infection and subsequently the development of premalignant dysplasias [61]. Intramuscular vaccination produces antibodies which are transferred to the site of the viral infection (cervix, vulva) by two mechanisms. First, IgG can traverse the epithelial layer into mucosal secretions through the neonatal Fc receptor that is expressed at the cervix [62] (Figure 1). Nevertheless, this mechanism may play a secondary role in vaccine protection, since L1 specific neutralizing antibodies in the cervical mucus of vaccinated women are 10 to 100 fold lower compared to serum concentrations [63]. The second and the most important mechanism includes the direct transfer of interstitial antibodies at the site of injury in the basal epithelial cell layer, thus blocking viral infection.

Although the development of HPV vaccines has been successful, the design of a vaccine against a wide range of HPV genotypes is difficult, since the L1 gene demonstrates significant variability among different HPV genotypes. On the other hand, the L2 gene is more conserved among HPV genotypes, thus it may also be considered for the design of future vaccines [64,65,66,67,68]. Antibodies against the L2 protein could provide a broad protection against various HPV genotypes, though the L2 protein alone cannot form VLPs and the antibody titers against it are considerably low [64,65,66,67,68]. Recent analyses have focused on the insertion of specific L2 peptides to bacteriophage coat proteins (MS2, PP7 or AP205) (45–46). The generation of chimeric bacteriophage coat proteins and their assembly into VLPs is a promising approach that provides strong protection against numerous HPV genotypes [69,70]. In particular, previous studies involved immunized mice with a MS2-L2 VLP that exposes a concatemer peptide comprising amino acids 17–31 from HPV16 L2 and a peptide containing amino acids 20–31 from HPV31 L2 in combination with a MS2-L2 VLP that exposes a consensus L2 peptide containing amino acids 69–86 [69,70]. This combination offered wide protection against various HPV genotypes including HPV 11, 16, 18, 31, 33, 35, 39, 45, 52, 53, 56, and 58 [69,70].

A special focus has been given to the HPV16 L2 region between amino acids 17 and 36, since it has been proposed that the respective region acts as a consensus sequence that could trigger the generation of antibodies against a broad range of HPV genotypes including HPV 6, 11, 16, 18, 26, 31, 33, 34, 35, 39, 43, 44, 45, 51, 52, 53, 56, 58, 59, 66, 68, and 73 [64]. In particular, the region between amino acids 17–36 from the HPV16 L2 gene is 90% similar to a consensus sequence of amino acid 17–36 from this region [64]. The immunization with the HPV16 L2 peptide (aa 17–36) seems to simulate the immunization with a consensus peptide, thus making the respective region a promising candidate for future vaccines [64]. As a result, the high conservation of the minor capsid protein L2 among different HPV genotypes constitutes for the respective protein an appealing target for the development of next generation vaccines that could provide protection against a wider range of HPV genotypes.

## 3. Genetic Variability of the HPV16 L1 Gene

The HPV16 L1 gene is located between nucleotides 5559 and 7154 and it encodes the major capsid protein L1, which is 531 amino acids in size. The L2 gene is located between nucleotides 4235 and 5656 and it encodes the minor capsid protein L2 of 473 amino acids in size. The HPV capsid upon progeny virion formation is assembled in a T = 7 icosahedral symmetry around viral DNA via L1–L2 protein interaction [71]. Cryo-electron microscopy reconstructions and antibody binding analyses showed that the major capsid protein forms 72 pentamers that comprise a total of 360 copies of the L1 molecule, while the minor L2 capsid protein is included into the L1 pentamers [72,73]. The L1–L2 protein interaction is hydrophobic. Specifically, the L2 protein is inserted into the central lumen of the L1 pentamer, whereas an N-terminal “external loop” of approximately 60 amino acids of L2 proteins seems to be disclosed on the capsid surface [74,75]

Intratypic HPV16 variants share more than 98% L1 sequence similarity with the reference sequence of the viral genome (reference HPV16 genome NC_001526) [72,76]. Considering the HPV16 L1 gene, specific nucleotide sequence variations have been associated with particular HPV16 lineages. More specifically, the A1–A3 sub-lineages harbor the T6861C variation; the A4 sub-lineage harbors the G7059A variation; the B lineage harbors 12 variations (G5697A, C5863T, T5910C, C6164A, T6246C, A6315G, C6558T, T6567A, G6720A, C6853T, C6969T, G6993A, G7059T); the C lineage harbors 15 variations (G5697A, C5863T, T5910C, C6164A, T6246C, A6315G, T6481C, C6558T, A6694C, G6720A, C6853T, C6864T, C6969T, G6993A, G7059T); the D lineage harbors 15 variations (G5697A, C5863T, T5910C, C6164A, T6246C, A6315G, C6558T, A6694C, G6720A, A6802T, C6853T, C6864T, C6969T, G6993A, and G7059T) [77].

The analysis of the L1 gene revealed that non-synonymous mutations were situated adjacent to the neutralizing epitopes, thus offering capsid L1 protein a considerable advantage to avoid immune response [78]. In particular, five L1 hypervariable surface loops of 10–30 amino acids in size [DC loop (aa 50–69), DE loop (aa 110–153), EF loop (aa 160–189), FG loop (aa 262–291) and HI loop (aa 348–396)] have been characterized through crystallography, and it has been proposed that they are recognized by human antibodies during immune response [79,80]. Although these hypervariable loops are exposed to circulating human antibodies, they have a crucial impact on capsid formation, as well. In particular, the DE and FG loops are responsible for the interaction of the L1 with the proline rich region of the L2, while the EF loop is essential for the generation of L1-L1 di-sulphidic bonds [81,82]. As a result, the amino-acid changes accumulated in the respective regions can influence not only the immunological host response but they can also modify the L1 protein structure and subsequently the efficient assembly of L1, L2 proteins into viral particles [83,84].

Research data collected from different studies revealed that numerous amino acid changes are found throughout the L1 ORF, while a considerable variability is detected near or within the region of the L1 gene that encodes for the respective immunodominant loops. In particular, a total of twenty-seven non synonymous mutations in L1 gene were recorded in patients from the Netherlands, while twenty one of these variations are located in the region that encodes for the immunodominant FG loop [85]. Nevertheless, the impact of these changes in immunological response is yet to be clarified. Interestingly, a previous analysis that was performed in India revealed seven non-synonymous nucleotide changes, namely C6163A (T202N), G6171A (A205T), C6240G (H228D), A6432G (T292A), G6693A (T379P) C6863T (P435L), and G7058T (L500F). The role of these sequence changes in the L1 epitopes were predicted in silico and their impact on immunogenicity was evaluated in vivo. According to these findings, it was proposed that L500F exhibits a 2.7-fold (*p* < 0.002) increase in antibody titer, while T379P presents a 0.4 fold decrease in antibody titer [47]. In addition, a previous analysis that was conducted in Morocco revealed a total of three non-synonymous nucleotide changes, A6694C (T389P; HI loop), G6800A (M424I;H2 helix) and G6818A (M430A; H2 helix), while 3D prediction models revealed that these changes do not affect the overall structure of the viral protein [48]. Moreover, it was found that the amino acid substitution T389P (HI loop) may influence the susceptibility of the respective L1 mutant to the vaccine induced immunity [48]. Finally, deletions and insertions have also been observed in the L1 sequence. In particular, an ATC insertion at position 6901 (H4 helix) and a GAT deletion at position 6950 (BC loop) are prevalent in all the examined cases from India, Morocco and Southwest China [47,48,86]. Nevertheless, 3D prediction models showed that these sequence changes do not modify the L1 protein structure [48].

A previous analysis in the Indian population revealed a total of 20 non synonymous mutations, nine of which are located in the immunodominant loops including N56T (BC loop), T176N, V178G, A179T, N181T/I (EF loop), T266A, N285T (FG loop) and T353P (HI loop).Three-dimensional prediction models suggest that the amino acid mutations L158F, A179T, K236T, T266A, S296R, T353P, S396P, and K454T might influence the stability of the protein structure, while the amino acid mutations N56T, H76Y, N92T, T176N, V178G, N181T/I, N285T, T389S, K443Q, and L474F might prevent the structure of the L1 monomer [87].

A more recent analysis that examined all the available complete and partial sequences of the HPV16 L1 gene demonstrated that the vast majority of nucleotide polymorphisms are accumulated in the DE (27.38%) and FG (31%) loops, while four mutations are frequently detected in other loops such as T176N (EF loop), N181T (EF loop), A266T (FG loop), and T353P/I/N (HI loop) [88]. Notably, the vast majority of L1 mutations located at the FG loop are found mainly in Europe, while mutations in the EF and HI loops are widely distributed in Asia. In contrast, sequence changes that were recorded in the DC and DE loops exhibit no significant association with the ethnicity of cases [88]. Considering research data from clinical and bioinformatics analyses, we postulated that different nucleotide changes in the L1 gene are prevalent in diverse geographic populations, thus affecting the sufficient immune response of hosts and consequently the development of cervical cancer. As a consequence, an extensive record of the most frequent amino acid substitutions of the HPV16 L1 protein could provide targeted improvements to vaccines specific to each geographic population.

## 4. Genetic Variability of the HPV16 L2 Gene

Although the L1 gene has been the focus of many studies, little is known concerning the variability of the HPV16 L2 gene. The role of the minor L2 capsid protein in viral pathogenicity is regarded as critical, since it is implicated not only in viral particle assembly and viral entry, but also ensures the efficient transport of viral DNA in the nucleus of infected cells and is involved in host immune response as well [76,89,90]. As was mentioned above, the N-terminal “external loop” of the L2 protein is exposed on the viral capsid surface, which is highly conserved, while it contains cross-neutralizing epitopes that are recognized by either neutralizing or cross-neutralizing antibodies [74,91,92]. Specific epitopes have been characterized, including the amino acid residues aa 17–36, aa 56–81, aa 65–81, and aa 108–120 [67,93,94,95,96,97]. However, the capsid L2 protein is regarded as immunologically subdominant to L1 [98,99].

Certain sequence variations within the HPV16 L2 gene are related to the different lineages. In particular, the A1–A3 sub-lineages harbor two variations (C4724T, G5235A); the A4 sub-lineage harbors four variations (G5044A, A5225C, C5368T, A5517C); the B lineage harbors 18 variations (T4280C, G4307A, G4427A, G4460A, T4599C, T4640C, T4643A, A4910T, G5141A, G5235A, A5289G, T5309C, G5378A, G5388A, T5402C, T5494C, G5505A, C5563G); the C lineage contains 22 variations (T4280C, G4427T, A4517G, T4544G, T4556G, T4599C, T4643A, C4853T, A4886G, G5141A, G5235A, A5258G, A5289C, T5309C, C5368T, G5378A, G5388A, T5402C, C5486T, T5494C, G5505A, C5563G); and the D lineage contains 28 variations (T4280C, G4427T, T4451C, G4460A, A4598C, T4599C, T4643A, A4886G, A4943G, A4949G, A4968G, A5033T, A5151C, G5235A, T5285A, A5294C, T5309A, T5367G, C5368T, G5378A, T5385G, G5388A, T5402C, T5474A, C5486T, T5494C, G5505A, C5563G) [77].

The analyses of the HPV16 L2 gene in clinical samples are rather limited. However, it has been observed that the L2 gene is polymorphic, whereas the region that encodes for the N-terminal domain of the L2 protein was highly conserved. In particular, a previous analysis that was carried out in patients from Southwest China revealed nine non-synonymous nucleotide variations, while no sequence changes in the residues 65–78 and 108–120 were detected [100]. In addition, in the Indian population, a total of 38 non-synonymous variations were observed in HPV16 positive malignant cases [101]. Interestingly, a more recent study in the Indian population revealed 16 novel amino acid substitutions in the HPV16 L2 protein with T245A, L266F, S378V and S384A found to be significantly associated with severe dysplasia [102]. Finally, a study that was performed in Thai women revealed a total of seven non-synonymous sequence variations, while no mutations were recorded in the N-terminal domain [103]. The L2 protein plays a pivotal role in viral function and structure. In addition, its N-terminal domain is highly conserved among different HPV16 strains.

## 5. The Frequency of non-Synonymous Changes within the HPV16 L1 and L2 Proteins across Different Populations

In order to perform a sequence analysis of publicly available sequence data, we searched the NCBI taxonomy database for Human papillomavirus type 16 (txid;333760) and retrieved 11,018 (10 June 2022) nucleotide records. These records were filtered based on the availability of country of isolation, thus resulting in 9658 sequences. These nucleotide sequences were subsequently scanned by the BLASTn algorithm [104] using the L1 and L2 CDS of the HPV16 type strain (NC_001526) as queries (e-value cutoff: 1e-10) resulting in 5013 and 4172 sequences, respectively. The sequences for each gene were translated and only those that had known amino acids in at least 90% of the reference protein length were kept, resulting in 3697 and 2059 sequences for the L1 and L2 genes, respectively. The final protein sequences were aligned using the MUSCLE multiple alignment algorithm [105] embedded in the Seaview software [106]. The consensus sequences were calculated for each of the final protein alignments with Jalview [107]. The number of sequences retrieved from the different geographic regions is presented in Table 1.

**Figure 2:**
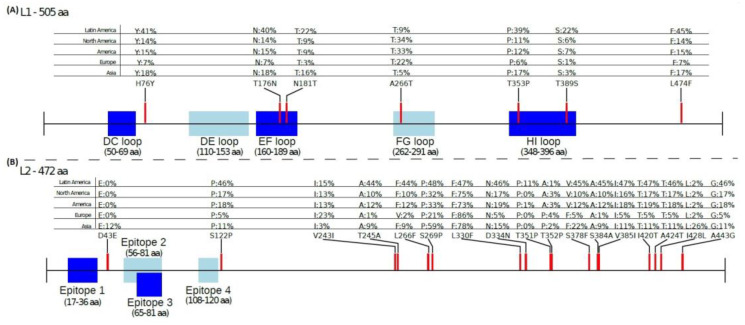
Mutation profile of HPV16 L1 and L2 proteins. The immunogenic regions of viral proteins are highlighted. (**A**) Seven amino acid mutations were detected in the L1 protein with high frequency in certain geographic areas. Five of these mutations (T176N, N181T, T266A, T353P, T389S) are located within the L1 hypervariable surface loops. (**B**) Seventeen amino acid mutations were found in the L2 protein with high frequency in various geographic populations. Two of these amino acid substitutions (D43E, S122P) are adjacent to the L2 specific epitopes.

A global amino acid mutation analysis of HPV16 L1 revealed a total of seven high frequency amino acid substitutions (H76Y, T176N, N181T, T266A, T353P, T389S, L474F) (63). The location of the respective mutations in the L1 protein as well as their distribution among different geographic locations is summarized in Table 2 and Figure 2. Interestingly, the mutations H76Y (N-terminal domain), T176N (EF loop), N181T (EF loop), T353P (HI loop), and L474F (C-terminal domain) are most frequently detected in South America followed by Asia, North America and Europe (Table 2, Figure 2). Moreover, the T266A mutation (FG loop) was most frequent in North America (34%), followed by Europe (22%), South America (9.1%) and Asia (5.3%) (Figure 2), while amino acid substitution T389S (HI loop) is most frequently found in South America (22%), followed by North America (6%), Asia (2.8%), and Europe (0.9%) (Table 2, Figure 2). The DC loop and the DE loop seems to be more conserved, as the frequency of amino acid substitutions at the corresponding positions was less than 0.1% in all geographic locations (see supplementary file).

The large-scale sequence analysis of the L2 gene revealed that the specific L2 epitopes (aa 17–36, aa 56–81, aa 65–81, aa 108–120) appear to be conserved among the different HPV16 isolates globally (Figure 2). However, a total of 17 amino acid mutations (D43E, S122P, V243I, T245A, L266F/V, S269P, L330F, D334N, T351P/S, T352P/A, S378V/F, S384A, V385I, I420T, A424T, I428L, A443G) were found with high frequency in the L2 protein (see supplementary file). The location of the respective mutations in the L2 protein and their distribution in various geographic areas is summarized in Figure 2. Although the N-terminal domain of the L2 protein was found to be highly conserved, two amino acid substitutions, D43E and S122P, exhibit high distribution among HPV16 isolates. These changes were found to be adjacent to L2 epitopes (Table 2, Figure 2). Considering their geographic distribution, it was revealed that D43E is observed exclusively in Asia (Table 2, Figure 2). In addition, the amino acid substitution S122P is most common in South America (46% of isolates), but is also observed in North America (17%), Asia (11%), and Europe (5.1%) (Table 2, Figure 2).

The large-scale sequence analysis of the L2 gene revealed that the specific L2 epitopes (aa 17–36, aa 56–81, aa 65–81, aa 108–120) appear to be conserved among the different HPV16 isolates globally (Figure 2). However, a total of 17 amino acid mutations (D43E, S122P, V243I, T245A, L266F/V, S269P, L330F, D334N, T351P/S, T352P/A, S378V/F, S384A, V385I, I420T, A424T, I428L, A443G) were found with high frequency in the L2 protein (see supplementary file). The location of the respective mutations in the L2 protein and their distribution in various geographic areas is summarized in Figure 2. Although the N-terminal domain of the L2 protein was found to be highly conserved, two amino acid substitutions, D43E and S122P, exhibit high distribution among HPV16 isolates. These changes were found to be adjacent to L2 epitopes (Table 2, Figure 2). Considering their geographic distribution, it was revealed that D43E is observed exclusively in Asia (Table 2, Figure 2). In addition, the amino acid substitution S122P is most common in South America (46% of isolates), but is also observed in North America (17%), Asia (11%), and Europe (5.1%) (Table 2, Figure 2).

The residual mutations of the HPV16 L2 protein are detected in the central region and the C-terminal domain (Figure 2). Notably, the amino acid substitutions T351P and I428L were characterized as population-specific. Specifically, the amino acid change T351P was predominant in South America (11.4%), but was rarely detected elsewhere. The amino acid mutation I428L was present almost uniquely in Asia, accounting for 26% of Asian isolates (Table 2, Figure 2), and the frequency of S269P and L330F was higher than that of the reference amino acid. In particular, at position 269, proline is detected more frequently than the reference amino acid serine. This phenomenon was only observed in Asian isolates (see supplementary file). Moreover, at position 330 phenylalanine is more common than the reference amino acid leucine in Europe, Asia, and North America (see supplementary file).

## 6. Discussion

Τhe US Food and Drug Administration (FDA) has approved a total of three prophylactic HPV vaccines based on the L1 protein. Until June 2020, 107 out of 194 WHO member states have started national HPV vaccination programs, whereas it has been estimated that in 2019 approximately 15% of girls and 4% of boys have been fully vaccinated [108]. The WHO Cervical Cancer Elimination Strategy aims to expand HPV vaccination to 90% of all adult women, double lifetime cervical screening to 70% and treatment of cervical intraepithelial lesions and cervical cancer to 90% by 2030 [109]. Currently available vaccines contain virus-like particles (VLPs) that are formed by the major capsid protein L1, imitating the structure of virions [50,51,52,53]. In recent years, a special focus has been given to the capsid protein L2 in the field of HPV vaccines. In the present review, we describe the variability in the HPV16 late genes (L1, L2) according to publicly available sequence data collected from different populations. Moreover, a large-scale sequence analysis was conducted in order to report the worldwide distribution of amino acid substitutions throughout HPV16 L1 and L2 proteins as well as to investigate whether these changes are found in specific immunodominant regions of viral capsid proteins.

Various amino acid substitutions have been recorded within or adjacent to the HPV16 L1 immunodominant loops [47,48,85,86,88]. However, the impact of these changes on immunological response has not been clarified thus fur. Although it has been proven that the amino acid changes L500F and T379P (HI loop) influence antibody titers in the Indian population, there are no available data concerning the role of these changes in different geographic populations [47]. Considering our large-scale analysis, we found seven amino acid substitutions (H76Y, T176N, N181T, T266A, T353P, T389S, L474F) that exhibit high frequency in the L1 protein globally. Five of these amino acid changes (T176N, N181T; EF loop), (T266A; FG loop), (T353P, T389S; HI loop) are of particular interest since they are located within the L1 hypervariable surface loops and they are preferentially detected in specific geographic areas [88]. In particular, the T176N, N181T, and T353P are predominant in America and Asia, the T266A was found in 22–33% of isolates from Europe and America, while the T389S is specific to the Americas (Table 2). It is worthy of note that recent 3D prediction models suggested that the amino acid substitutions T176N, N181T and T389S might be involved in stabilization of the L1 monomer structure, whereas the amino acid changes T266A and T353P seem to affect the stability of the L1 pentamer [87]. In particular, the T266A mutation leads to the loss of two hydrogen bonds with the amino acids at positions 360 and 361 of the adjacent chain, while the amino acid substitution T353P results in the loss of one hydrogen bond with the amino acid at position 26 of the adjacent pentamer [87]. The impact of these changes on the host-immune response requires further investigation, while more analyses are necessary to better assess whether these mutations enable an escape from L1 vaccines.

On the other hand, the L2 gene is considered as a polymorphic region, although its N-terminal domain is highly conserved [100,101,103]. Our large-scale analysis confirmed these observations. Moreover, we detected a total of 17 L2 amino acid substitutions that are frequently found in various geographic locations. Interestingly, two of these changes (D43E, S122P) were found adjacent to L2 epitopes and they had high frequency in specific geographic regions. In particular, the D43E is highly specific to Asians, while the S122P is predominant in specific geographic regions, including South America (46%), North America (17%), Asia (11%), and Europe (5.1%) (Table 2). More recent findings suggested that the S122P is common to the epitope region recognized by MHC-I and MHC-II, according to B and T cell epitope prediction models [102]. However, the role of these changes in the tertiary structure of viral proteins as well as their impact on immunological response requires further investigation.

It is of note that the amino acid changes that were found next to or within the immunodominant regions of L1 and L2 proteins are highly distributed in specific geographic areas. This variability might influence the potential of certain HPV16 strains to avoid immunological response in some populations, providing HPV16 strains with a selective advantage to establish persistent infection, thus leading to more severe dysplasia. However, further analyses of these amino acid changes need to be performed in order to confirm this hypothesis.

Highly conserved regions of the HPV16 L1, L2 proteins have been extensively characterized, including the DC loop, DE loop of the L1 protein, the L2 specific epitopes and the N-terminal domain of the L2 protein [48,88,100,101,103]. The high conservation of these regions might aid the virus to maintain the integrity of the viral capsid. Moreover, it is concluded that these highly conserved regions might be suitable targets for future vaccines. It is important to underline that HPV16 L2 amino acid region 17–36 is highly conserved not only among the different HPV16 isolates, but also among different HPV genotypes [64,110]. Therefore, L2 should be considered as an appealing target for future broad-range vaccines [64,110]. A limitation of the present analysis was the small number of the available HPV16 L1 and L2 sequences in certain geographic regions. Hence, more studies in the future with more available data would be essential to provide a thorough overview of L1 and L2 gene diversity in these geographic areas, underlying mutations that could change the L1 and L2 protein structure and consequently influence the specific immunodominant regions.

## 7. Conclusions

To the best of our knowledge, this is the first extensive literature review and large-scale sequence analysis concerning the global variability of both HPV16 L1 and L2 genes. We presented important data concerning the variability of the late region of HPV16 DNA. Our observations suggest that the high prevalence of certain amino acid substitutions within or adjacent to specific immunodominant regions might influence the stability of the viral capsid as well as modify the ability of HPV16 in evading host—immune response in specific populations. The extensive mapping of amino acid substitutions in the HPV16 L1 and L2 epitopes should be cautiously considered in a given population.

## Figures and Tables

**Table 1:** Sequences retrieved from the different geographic regions.

Gene	Geographic Location	Sequences (n)
L1	Africa	1
L1	Asia	356
L1	Europe	456
L1	America	2884
L1	South America	143
L1	North America	2741
L2	Africa	0
L2	Asia	233
L2	Europe	175
L2	America	1651
L2	South America	105
L2	North America	1546

**Table 2:** Distribution of amino acid substitutions in the L1 and L2 proteins in various geographic regions.

					Total Data				Europe				Asia				Total America				North America				South America			
		Pos	Ref Pos	Ref aa	Major aa	aa %	Subs aa	aa %	Major aa	aa %	Subs aa	aa %	Major aa	aa %	Subs aa	aa %	Major aa	aa %	Subs aa	aa %	Major aa	aa %	Subs aa	aa %	Major aa	aa %	Subs aa	aa %
**L2**	**w**	**43**	43	D	D	98.6	E	1.4	D	100	-	0	D	88	E	12	D	100	-	0	D	100	-	0	D	100	-	0
		**122**	122	S	S	83.5	P	16	S	94.9	P	5.1	S	88	P	11.2	S	81.6	P	18	S	83	P	16.6	S	54.3	P	45.7
**L1**		**176**	176	T	T	85.8	N	14	T	93.2	N	6.8	T	82.3	N	17.7	T	85.1	N	15	T	86	N	13.6	T	60.1	N	39.9
	**EF loop**	**181**	181	N	N	90.8	T	9.2	N	96.7	T	3.3	N	83.7	T	16	N	90.7	T	9.3	N	91	T	8.65	N	78.3	T	21.7
	**FG loop**	**266**	266	T	A	71	T	29	A	77.9	T	22.1	A	94.7	T	5.3	A	67.1	T	33	A	66	T	34.2	A	90.9	T	9.1
		**353**	353	T	T	88	P	12	T	94.1	P	5.9	T	83.1	P	16.9	T	87.6	P	12	T	89	P	10.8	T	60.6	P	39.4
	**HI loop**	**389**	389	T	T	94.3	S	5.7	T	99.1	S	0.9	T	96.9	S	2.8	T	93.2	S	6.8	T	94	S	5.98	T	78.2	S	21.8

## Data Availability

Data is included in the manuscript.

## Supplementary Materials

The following supporting information can be downloaded at: https://www.mdpi.com/article/10.3390/v15010141/s1, Supplementary file: Distribution of amino acid substitutions in the complete amino acid sequence of HPV16 L1 and L2 proteins in different geographic locations.
